# Sequence of Closely Related Plasmids Encoding *bla*_NDM-1_ in Two Unrelated *Klebsiella pneumoniae* Isolates in Singapore

**DOI:** 10.1371/journal.pone.0048737

**Published:** 2012-11-06

**Authors:** Ying-Tsong Chen, Ann-Chi Lin, L. Kristopher Siu, Tse Hsien Koh

**Affiliations:** 1 Institute of Molecular and Genomic Medicine, National Health Research Institutes, Zhunan, Taiwan; 2 Institute of Genomics and Bioinformatics, National Chung Hsing University, Taichung, Taiwan; 3 Biotechnology Center, National Chung Hsing University, Taichung, Taiwan; 4 National Institute of Infectious Diseases and Vaccinology, National Health Research Institutes, Miaoli, Taiwan; 5 Graduate Institute of Basic Medical Science, China Medical University, Taichung, Taiwan; 6 Department of Pathology, Singapore General Hospital, Singapore, Singapore; Cornell University, United States of America

## Abstract

**Background:**

Spread of the *bla*_NDM-1_ gene that encodes the New Delhi metallo-β-lactamase (NDM-1) in Enterobacteriaceae is a major global health problem. Plasmids carrying *bla*_NDM-1_ from two different multi-drug resistant *Klebsiella pneumonia* isolates collected in Singapore were completely sequenced and compared to known plasmids carrying *bla*_NDM-1_.

**Methodology/Principal Findings:**

The two plasmids, pTR3 and pTR4, were transferred to *Escherichia coli* recipient strain J53 and completely sequenced by a shotgun approach using 3-kb paired-end libraries on 454. Although the *K. pneumoniae* strains were unrelated by molecular typing using PFGE and MLST, complete sequencing revealed that pTR3 and pTR4 are identical. The plasmid sequence is similar to the *E. coli* NDM-1-encoding plasmid p271A, which was isolated in Australia from a patient returning from Bangladesh. The immediate regions of the *bla*_NDM-1_ gene in pTR3/4 are identical to that of p271A, but the backbone of our plasmid is much more similar to another IncN2 plasmid reported recently, pJIE137, which contained an additional 5.2-kb CUP (conserved upstream repeat) regulon region in comparison to p271A. A 257-bp element bounded by imperfect 39-bp inverted repeats (IR) and an incomplete version of this element flanking the 3.6-kb NDM-1-encoding region were identified in these plasmids and are likely to be the vestiges of an unknown IS.

**Conclusions:**

Although the hosts are not epidemiologically linked, we found that the plasmids bearing the *bla*_NDM-1_ gene are identical. Comparative analyses of the conserved NDM-1-encoding region among different plasmids from *K. pneumoniae* and *E. coli* suggested that the transposable elements and the two unknown IR-associated elements flanking the NDM-1-encoding region might have aided the spreading of this worrisome resistance determinant.

## Introduction

The NDM-1 carbapenemase gene has become an important resistant determinant in Gram-negative bacteria [1], [2]. NDM-1 is able to hydrolyze almost all β-lactam antibiotics and when combined with other resistance mechanisms, renders the host bacterium resistant to almost all antibiotics [3], [4]. The rapid spread of these multidrug resistant strains is now a matter of global concern.

Initially, plasmids encoding *bla*_NDM-1_ were observed in *Klebsiella pneumoniae* and *Escherichia coli* [5]. These plasmids can conjugatively transfer into other species. The concern in India is the heavy contamination of this gene in seepage water with the possibility of spread in the community [6]. Travelers may be colonized with NDM-1 producing strains in the gut resulting in spread of the gene to different countries [7], [8]. The *bla*_NDM-1_ gene has been identified on different plasmids types that vary in length from ∼50 to 300 kb [9], [10]. In addition, *bla*_NDM-1_ has recently been identified in the chromosome of *Acinetobacter baumannii* [11]. The resistance gene was also reported recently in other bacterial species, such as *Vibrio cholerae* [6]. Thus, the rapid global spread of *bla*_NDM-1_ may not be explainable by a single mechanism.

In this study, the complete sequence of conjugatively transferrable plasmids encoding NDM-1 from two *K. pneumoniae* clinical isolates were determined to investigate the genetic basis of the resistance gene. Comparative analyses were carried out with existing sequences to investigate the molecular mechanism underlying the spread of *bla*_NDM-1_ in bacteria.

## Materials and Methods

### Patients’ Characteristics

Patient 1 was a 36 year old male Chinese local with lymphocytic meningitis of undetermined cause. He had no recent travel history in the last year. Multi-drug resistant *K. pneumoniae* 43320 was a clinical isolate from urine during his rehabilitation 3 months after admission. He had a single spike of temperature but was not septic. He recovered without specific antimicrobial treatment. Patient 2 was a 22 year old male foreigner from Vietnam admitted 2 months after Patient 1 to a different ward in the same hospital with T4 hemangioma with cord compression. Multi-drug resistant *K. pneumoniae* 44951 was a clinical isolate from urine 8 days after admission and 10 days from the isolate from patient 1. As this was a catheter specimen, it was considered as insignificant and no specific antimicrobial treatment was given. Although their hospital stays overlapped, there was no obvious epidemiological link between the 2 patients.

### Antimicrobial Susceptibility Testing

The MICs of 15 antimicrobial agents were determined using the broth microdilution test according to the recommendations of the Clinical and Laboratory Standards Institute [12].

### General characteristics of NDM-1 Carrying K. pneumoniae

The 2 carbapenem resistant *K. pneumoniae* were confirmed to be carrying *bla*_NDM-1_ by PCR and subsequent sequencing according to previously published primers for *bla*_NDM-1_ [13]. Plasmid conjugation was performed using *E. coli* J53 azide resistant strain as recipient [14]. Briefly, recipients and *bla*_NDM-1_ carrying *K.* *pneumoniae* were separately inoculated into brain heart infusion broth (Oxoid Ltd., Basingstoke, England) and incubated at 37°C for 4 h. They were then mixed at a ratio of 1∶10 (Donor:Recipient by volume) for overnight incubation at 37°C. A 0.1-ml volume of the overnight broth mixture was spread onto a MacConkey agar plate containing sodium azide (100 µ/mL) and imipenem (2 µg/mL).

### Molecular Typing for NDM-1 Carrying K. pneumoniae and their Transconjugants

Multilocus sequencing typing (MLST) and Pulsed field gel electrophoresis (PFGE) were performed for both *bla*_NDM-1_ carrying *K. pneumoniae* strains. Sequences of seven housekeeping genes for MLST were obtained according to Diancourt *et al*., Sequences were compared with those on the MLST web site (http://www.pasteur.fr/recherche/genopole/PF8/mlst/Kpneumoniae.html) developed by Diancourt et al., [15] and alleles and sequence types (STs) were assigned accordingly. If there was a difference in two or more alleles the strains were considered to be unrelated.

For PFGE, DNA was prepared as described previously [16]. The restriction enzyme *Xba*I (New England Biolabs, Beverly, MA, USA) was used at the manufacturer’s suggested temperature. Restriction fragments were separated by PFGE in 1% agarose gel (Bio-Rad, Hercules, CA, USA) in 0.5×TBE buffer (45 mM Tris, 45 mM boric acid, 1.0 mM EDTA, pH8.0) for 22 h at 200 V at a temperature of 14°C, with ramped times of 2 to 40 s using the Bio-Rad CHEF MAPPER apparatus (Bio-Rad Laboratories, Richmond, CA, USA). Gels were then stained with ethidium bromide and photographed under ultraviolet light. The resulting genomic DNA profiles, or “fingerprints”, were interpreted according to established guidelines [17]. Plasmid replicon typing was performed for transconjugants [18].

### Plasmid Sequencing

DNA sequencing of the NDM-1-carrying plasmids was performed with a whole genome shotgun approach using 3-kb paired-end libraries [19]. DNA fragments of about 3-kb in length were recovered after hydrodynamic shearing and purified using size exclusion beads (AMPure, Agencourt). The DNA fragments were subsequently linked to adaptors and circularized, then sheared again by nebulization. The resulting nucleotide fragments containing the adaptor were specifically purified, then ligated to oligomers for PCR amplification. The following emulsion-based clonal amplification (emPCR) was performed following standard 454 pyrosequencing protocols. Sequencing was performed using a 454 GS Jr (454 Life Sciences, Branford, CT, USA). The complete nucleotide sequences of plasmid pTR3 and pTR4 have been submitted to GenBank and assigned sequence accession number JQ349086 and JQ349085.

### Bioinformatics Analysis

De-novo sequence assembly was performed on a computer workstation using the 454 Newbler, which automatically detects long paired-end reads (Version 2.6, 454 Life Sciences, Branford, CT, USA). The contigs were manually inspected and reassembled using the Phred/Phrap/Consed [20]. Annotation of the plasmid was manually curated after performing automatic annotation on the RAST Server [20]. Insertion sequences and transposons were further annotated using ISfinder (http://www-is.biotoul.fr) [21].

## Results

### Antimicrobial Susceptibility Testing Results for NDM-1 Carrying K. pneumoniae and their Transconjugants

Antimicrobial susceptibility testing results showed that *bla*_NDM-1_ carrying *K. pneumoniae* clinical isolates, 43320 and 44951, from patient 1 and 2 respectively, were resistant to all tested antibiotics (Table 1). Addition of a β-lactamase inhibitor did not increase the susceptibility to β-lactams. The *E. coli* transconjugants, TCJ-P1 and TCJ-P2, respectively from 43320 and 44951 had a different resistance profile when compared with the corresponding clinical isolates but were still resistant to all β-lactams except aztreonam. Reduced MICs for imipenem and meropenem were observed in both transconjugants. Both transconjugants were susceptible to non-β -lactam antibiotics including ciprofloxacin, gentamicin, tetracycline and trimethoprim/sulfamethoxazole. PFGE of 43320 and 44951 showed that they were unrelated with more than six bands difference. MLST indicated that 43320 and 44951 belonged to ST273 and ST1 respectively (data not shown). The plasmid incompatibility typing initially was positive for a product with a size consistent with the PCR product for IncN. However, subsequent sequencing of the PCR products showed unrelated sequences for a putative IS*911* transposase *orf*A with KpLE2 phage-like element.

**Table 1:** Antimicrobial susceptibility test among *bla*_NDM-1_ carrying isolates and their transconjugants.

Antibiotics	Minimal inhibitory concentration (µg/µl)				
	43320	TCJ-P1		44951	TCJ-P2
Ampicillin	≥32	≥32		≥32	≥32
piperacillin/tazobactam	≥128	≥128		≥128	≥128
Cefazolin	≥32	≥32		≥32	≥32
Cefpodoxime	≥64	≥64		≥64	≥64
Cefoxitin	≥128	≥128		≥128	≥128
Cefotaxime	≥128	≥128		≥128	≥128
Cefotaxime/clavulanate	≥128	≥128		≥128	64
Ceftazidime	≥128	≥128		≥128	≥128
Ceftazidime/clavulanate	≥128	≥128		≥128	≥128
Ceftriaxone	≥128	≥128		≥128	≥128
Cefepime	≥32	≥32		≥32	≥32
Aztreonam	≥32	≤4		≥32	≤4
Imipenem	16	8		16	4
Meropenem	≥16	4		≥16	2
Ciprofloxacin	≥4	≤1		≥4	≤1
Gentamicin	≥32	≤4		≥32	≤4
Tetracycline	≥32	≤4		≥32	≤4
†Trimethoprim/sulfamethoxazole	≥8	≤1		≥8	≤1

* 43320 and 44951, clinical isolates from patient 1 and 2 respectively; TCJ-P1 and TCJ-P2, transconjugants from 43320 and 44951 respectively.

† The MIC is presented according to the concentration of trimethoprim.

### Sequence Annotation and Comparison of the Two bla_NDM-1_ Plasmids

**Figure 1:**
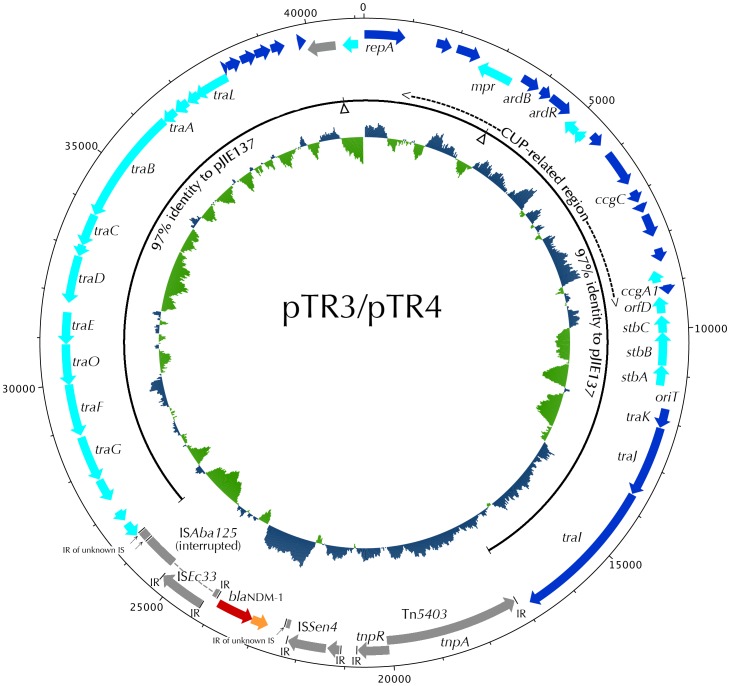
Circular map of plasmid pTR3 and pTR4. The open reading frames are marked along the map by arrows and significant ones are labeled. The *bla*_NDM-1_ gene (red) is located in a region with several transposon/IS-related genes (gray). The region corresponding to the IncN2 backbone of pJIE137 is indicated by a black line. Positions of the two resistance regions (a class 1 integron/Tn and a complex IS*Ecp1*-*bla*_CTX-M-62_ transposition unit) present in pJIE137 but missing in pTR3/4 are marked by the arrowheads. The CUP-related region between *repA* and *stbABC* is missing in p271A. G+C% are shown in the inner circle.

**Figure 2:**
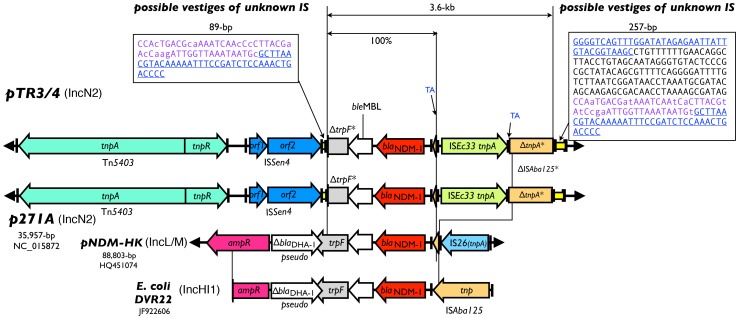
Schematic diagram of the NDM-1 region of pTR3 and pTR4, compared to those from the other known plasmids. The *bla*_NDM-1_ (red), and nearby IS elements (various colors) are shown. ORFs are depicted with arrows and the IRs were depicted by short vertical bars. The regions corresponding to possible vestiges of unknown IS identified in pTR3/4 and p271A are marked by yellow rectangles. Nucleotide sequences of the two regions are shown in the boxes, of which the 39-bp putative IRs are underlined. Corresponding repeat sequences in the boxes are shown in the same color. Differences are shown in lower case.

Complete sequencing was performed for the two circular *bla*_NDM-1_ plasmids, pTR3 and pTR4, respectively from 43320 and 44951. The results of the assemblies of the two plasmids based on 454 reads were almost identical, in that only seven locations of indels were found between pTR3 and pTR4. Subsequent sequence verifications by Sanger reads have shown that the two 41,188-bp plasmids are completely identical. Subsequent annotation of the plasmid, designated as pTR3/4, revealed 52 CDS (Figure 1). The nucleotide sequence of pTR3/4 is very similar to p271A, a 35,957-bp NDM-1 plasmid identified in *E. coli* 271 from a patient following medical transfer from a hospital in Bangladesh to Australia (GenBank: accession no. NC_015872 and [22]. Sequence comparison indicates the major difference between pTR3/4 and p271A is an additional 5.2-kb region containing hypothetical protein genes between *repA* and the *stbABC* genes in our plasmid. The genes resident in the 5.2-kb region represent the unique CUP (conserved upstream repeat)-controlled regulon of plasmid pJIE137, a 58,107-kb *bla*_CTX-M-62_-encoding plasmid from *K. pneumoniae* JIE137 identified in Australia [23]. Similar to p271A, and JIE137, pTR3/4 also have a backbone organization similar to the IncN plasmid R46. In addition, the conserved *repA* in these plasmids are unrelated to the IncN plasmids [23]. Since the 5.2-kb CUP region is missing in p271A, the backbone of pTR3/4 is more closely related to pJIE137. Comparative genomics studies revealed that, apart from a 9-kb region containing the *bla*_NDM-1_ gene in pTR3/4 (Figure 2) and two resistance regions (a class 1 integron/Tn and a complex IS*Ecp1*-*bla*_CTX-M-62_ transposition unit) in pJIE137, the backbone sequences of pTR3/4 and pJIE137 are 97% identical (Figure 1).

### Sequence Comparison of the Immediate Region Near bla_NDM-1_

The immediate flanking regions of *bla*_NDM-1_, including the *ble*_MBL_ bleomycin-resistance protein gene, the *trpF* pseudogene, the nearby IS*Aba125* (interrupted), IS*Ec33*, IS*Sen4* and Tn*5403* are identical in pTR3/4 and p271A (Figure 2). Upstream of *bla*_NDM-1_ is a short fragment corresponding to the left extremity of an IS*Aba125*. When compared with the *E. coli* DVR22 sequence from Spain [GeneBank accession no. JF922606; [24]], it is apparent that the IS*Aba125* was interrupted by insertion of the IS*Ec33*, which produces a 2-bp target duplication (TA) during the event (Figure 2, marked blue adjacent to the IS*Ec33* IRs). When compared with the DVR22 sequence, the IS*Aba12*5 in pTR3/4 and p271A were all interrupted at the same position (…TATCÂ). A detailed analysis of the sequences adjacent to the interrupted IS*Aba125* revealed a 257-bp element bounded by a pair of 39-bp inverted repeats (blue and underlined in Figure 2) [23]. An 89-bp incomplete version, which consists of only the right end of the 257-bp element (11 differences in 89-bp, shown in lowercase in Figure 2), including one of the 39-bp IR, was found at the other side of the NDM-1 region. The 39-bp imperfect IR (6 differences) associated with these elements are different from the 38-bp IR of the nearby Tn*5403*. Compared to pNDM-HK and DVR22, the *trpF* pseudogenes in pTR3/4 and p271A were all truncated by this IR-associated element, of which the left extremity is further truncated by the IS*Sen4*. We hypothesize that the 257-bp element and the 89-bp element (marked yellow and sequence shown in the boxes in Figure 2) may be the remains of an unknown IS that transposed into a progenitorial sequence similar to that of the *E. coli* DVR22.

## Discussion

A diversity of *bla*_NDM-1_ plasmids have been observed in different published studies. Although plasmid carrying *bla*_NDM-1_ was first described in *K. pneumoniae*, the plasmid incompatibility type was not determined in that study [13]. Subsequent studies revealed plasmid scaffolds of IncL/M type in Hong Kong [14], IncA/C type in Japan [25], IncN2 type from Bangladesh [22], IncF, type in India [26], and recently IncP type in China [9]. In this study, two isolates carrying *bla*_NDM-1_ on plasmids similar to IncN2 were identified in two patients who were not epidemiologically linked to each other (Figure 1). These two isolates were resistant to all tested antibiotics (Table 1). Transconjugants showed resistance only to all tested β-lactams except aztreonam. Thus, chromosomal and/or other plasmid-mediated resistance to antibiotics other than β-lactams were very likely present in their parental strains. Complete sequencing showed that although the parent isolates were unrelated based on molecular typing using PFGE and MLST, the plasmid carrying the *bla*_NDM-1_ is the same.

Overall, the *bla*_NDM-1_–carrying plasmid pTR3/4 is very similar to the *bla*_NDM-1_-encoding plasmid p271A from *E. coli* strain 271 collected in 2009 from a patient from Bangladesh [22]. A recently reported *bla*_CTX-M-62_-containing plasmid, pJIE137, also possesses a similar backbone to p271A, but carries a 5.2-kb CUP regulon region in addition [23]. These plasmids are referred to as an IncN2 subgroup which have a backbone similar to the IncN plasmid R46 but a *repA* gene unrelated to incN plasmids [22], [23]. Plasmid pTR3 and pTR4 also possesses the CUP regulon region and are closely related to these plasmids, especially with respect to the pJIE137 backbone. The discovery of pTR3/4 adds to the IncN2 subgroup of plasmids that cannot be classified using current PCR-based surveys. It appears that the resistance genes were acquired by this plasmid backbone and have been spreading to different locations in the world. Comparison with pJIE137 revealed that the 9,180-bp *bla*_NDM-1_-containing insert region in pTR3/4, as depicted in Figure 2, was bounded by the outermost IR of Tn*5403* and the 257-bp element at the other end. It had been proposed that in p271A the formation of this insert region was probably a result of progressive insertions and deletions of transposons in the *fipA* gene in the pJIE137 backbone or insertion of the entire region as a hybrid transposon created elsewhere [23]. The loss of the 5.2-kb CUP regulon region in p271A, on the other hand, may be explained by recombination between CUP repeats [23], [27]. It is likely the 9,180-bp bp *bla*_NDM-1_ containing region may have been inserted and settled in the pJIE137-like backbone form pTR3/4, while subsequently loss of the CUP regulon region in pTR3/4 resulted in p271A. The unknown IR-associated elements associated with *bla*_NDM-1_ and the interrupted IS*Aba125* was first described in a comparative analysis between p271A and pJIE137 [23]. In our analysis, the sequence associated with the IR in the 89-bp element is 88% identical to that bounded by the IRs in the 257-bp element (11 in 89 nucleotide positions, colored purple in Figure 2). While we think these elements may be the remains of an unknown IS, it is also possible that they are from related but different IS. The similarities between these IRs and the 38-bp IR from the nearby Tn*5403* (50% and 53% identity in 38 nucleotide positions) have also been reported [23]. When comparing the sequence homology to other NDM-1-encoding plasmids, the 257-bp and 89-bp elements comprised by the remains of unknown IS are very likely the factor to facilitate the transposition of *bla*_NDM-1_ from the progenitor sequence in *E. coli* DVR22 instead of pNDM-HK. This finding suggests that different IS elements increase the efficiency of resistance gene spreading.

In the present study, we have observed that the transmission of *bla*_NDM-1_ could be achieved by incorporation of transposable elements prior to plasmid spreading. This dual method for spreading may increase the incidence in the prevalence of bacteria carrying *bla*_NDM-1_. Since transposition could have occurred by incorporation of the resistance gene into the plasmid or chromosome, a diversity of Inc plasmid types with *bla*_NDM-1_ is to be expected and should also be identified in bacteria other than *K.* *pneumoniae*. In conclusion, we have identified a plasmid spreading in *K. pneumoniae* strains that are not epidemiologically linked. An unknown insertion element may be responsible for the mobilization of *bla*_NDM-1_ into an IncN2 plasmid backbone similar to pJIE137 and p271A. Comparative genomic studies on the IncN2 plasmids have revealed interesting features related to the accumulation and molecular evolution mechanisms of the plasmid scaffold.
